# A qualitative study of high-performing primary care practices during the COVID-19 pandemic

**DOI:** 10.1186/s12875-021-01589-4

**Published:** 2021-11-25

**Authors:** Stephanie L. Albert, Margaret M. Paul, Ann M. Nguyen, Donna R. Shelley, Carolyn A. Berry

**Affiliations:** 1 Department of Population Health, NYU Grossman School of Medicine, 180 Madison Avenue, 2nd Floor, New York, NY 10016 USA; 2 Center for State Health Policy, Rutgers, The State University of New Jersey, 112 Paterson Street, New Brunswick, NJ 08901 USA; 3 Global Center for Implementation Science, Department of Policy and Public Health Management, New York University School of Global Public Health, 665 Broadway, 8th Floor, New York, NY 10012 USA

**Keywords:** Primary care, COVID-19, Chronic disease, Preventive care, Qualitative

## Abstract

**Background:**

Primary care practices have remained on the frontline of health care service delivery throughout the COVID-19 pandemic. The purpose of our study was to understand the early pandemic experience of primary care practices, how they adapted care processes for chronic disease management and preventive care, and the future potential of these practices’ service delivery adaptations.

**Methods:**

We interviewed 44 providers and staff at 22 high-performing primary care practices located throughout the United States between March and May 2020. Interviews were transcribed and coded using a modified rapid assessment process due to the time-sensitive nature of the study.

**Results:**

Practices reported employing a variety of adaptations to care during the COVID-19 pandemic including maintaining safe and socially distanced access through increased use of telehealth visits, using disease registries to identify and proactively outreach to patients, providing remote patient education, and incorporating more home-based monitoring into care. Routine screening and testing slowed considerably, resulting in concerns about delayed detection. Patients with fewer resources, lower health literacy, and older adults were the most difficult to reach and manage during this time.

**Conclusion:**

Our findings indicate that primary care structures and processes developed for remote chronic disease management and preventive care are evolving rapidly. Emerging adapted care processes, most notably remote provision of care, are promising and may endure beyond the pandemic, but issues of equity must be addressed (e.g., through payment reform) to ensure vulnerable populations receive the same benefit.

## Background

Primary care practices have remained on the frontline of health care service delivery throughout the COVID-19 pandemic [1]. Further, the morbidity and mortality of COVID-19 with respect to underlying risk factors has particularly underscored the connection between chronic and infectious disease and highlighted a nationwide need for enhanced chronic disease prevention and treatment, both during and after the pandemic [2]. A small and growing body of research has documented significant gaps in chronic disease management and preventive care as a direct result of the COVID-19 pandemic, including less HbA1c monitoring for diabetes control [3], sharp declines in screening for breast cancer [4], and decrease in regularly scheduled general medical appointments [5]. Missed opportunities for testing and screening may be deleterious to health and result in increased medical costs [6].

The foundation for many changes in the early stages of the pandemic and beyond was a dramatic increase in the use of telehealth whenever possible [7–10]. Throughout the US, telehealth was successfully used for a variety of core primary care functions related to chronic disease management and preventive care during the early pandemic period. Practices established processes to remotely monitor high-risk patients (e.g., use of a registry to identify patients in need of a virtual appointment to manage hypertension) [11]. They also offered virtual group visits on healthy lifestyle changes for patients with diabetes [12]. Further, practices modified screening prevention practices such as using a medication refill request to schedule a virtual visit and assess medication adherence [13] and conducted pre-visit screening virtually in preparation for in-person visits [10].

The purpose of our study was to understand the experiences of primary care practices as they adapted during the early stages of the pandemic, particularly with respect to the rapid shift to telehealth, the care and management of patients with chronic disease, routine screening and preventive care of all patients, and the significance of these factors on access to care and equity. Our goal was to also describe the future potential of emerging service delivery adaptations among the high-performing practices in our study. To the authors’ knowledge there are only two peer-reviewed studies that have qualitatively assessed lessons learned from primary care practices during the early pandemic in the United States. The first focused only on practices in New York City [14], while the second concentrated specifically on the transition to telemedicine in Northern California clinics [15]. Our study is novel because it uses a sample of high-performing practices located throughout the US to understand broadly how they managed their patients’ healthcare during the early months of the COVID-19 pandemic. This gave us an opportunity to learn from practices that are likely among the most capable and qualified to adapt to an unprecedented public health crisis. We interviewed providers and staff members most closely involved in quality improvement at each practice and discussed specific ways in which they altered daily operations in response to the crisis. Finally, in this paper we discuss ways in which context may affect the various interventions discussed in order to inform future practice.

## Methods

This study is part of a larger effort to develop and validate a tool that aims to assess primary care structures and processes that are predictive of high-quality care and patient outcomes. All study protocols and procedures were approved by the NYU Grossman School of Medicine Institutional Review Board. All methods were performed in accordance with the relevant guidelines and regulations and all participants provided informed consent to participate.

### Sampling and recruitment

Our sampling frame included 266 adult primary care practices within four practice networks: Distributed Ambulatory Research in Therapeutics Network (DARTNet), NYC Department of Health and Mental Hygiene’s Bureau of Equitable Health Systems (BEHS), NYU Langone Faculty Group Practice, and OCHIN (not an acronym). Together, these practice networks represent geographically and demographically diverse primary care practices. In order to be eligible, practices had to be a primary care clinic, 70% of patient encounters had to be with patients aged 18 or older, and practices had to be “high performing”. We defined “high performing,” based on the Million Hearts initiative [16], as achieving good patient outcomes on two or more quality indicators (i.e., 70% of eligible patients prescribed aspirin use, 70% of patients with a diagnosis of hypertension met targets for blood pressure control (< 140/90), 70% of eligible patients prescribed statin therapy, and 90% of eligible patients with HbA1c < 9). The performance criterion was included because the larger study aimed to understand what structures and processes are important contributors to quality outcomes with respect to chronic disease management and preventive care.

We utilized a multi-stage recruitment process. First, practice networks shared de-identified practice information including location, size, and ownership for all eligible practices with the NYU study team. The study team then prioritized practices for recruitment in an effort to maximize variation. The goal was to recruit 30 diverse practices across the country. Next, practice network staff contacted individual practices to explain the study and secure agreement to participate. Networks provided practice contact information to the study team once clinics expressed interest in contributing to the study. Last, the research team contacted clinics directly to identify two participants from each clinic to interview (i.e., lead medical provider and quality improvement specialist/office manager) and to schedule interviews. We recruited 29 practices, and 22 participated in the study. Five practices declined to participate due to COVID-related scheduling challenges, one declined because of internal staffing changes, and one practice merged with another practice. We interviewed 44 individuals in 22 practices.

### Data collection

All interviews about the impact of COVID-19 took place between March and May 2020 and were conducted remotely using video conference due to COVID-19. The research team had developed a semi-structured interview guide for the larger study. As soon as COVID-19 emerged as a national public health crisis, the guide was modified to include a COVID-19 module including the following questions: (1) *What are you doing to continue delivering care during COVID-19?; (2) How are you managing patients for chronic care, such as hypertension and diabetes and preventive care?; and, (3) What care are you not able to provide as a result of COVID-19? Are there certain groups of patients that are primarily impacted? How are you providing care to these groups?* Practices that participated prior to the development of these questions were asked to participate in a brief follow-up interview examining just the COVID-specific questions. Two interviewers conducted each interview, and the pandemic-specific questions lasted between 20 and 30 min. Participants received a $150 honorarium (up to $300 per practice) for their time and effort. All interviews were recorded and transcribed by a professional transcription service, and the research team checked transcripts for accuracy prior to data analysis.

### Data analysis

The research team employed a rapid assessment process when analyzing these data due to the time-sensitive nature of the study [17]. We created a codebook in which each interview guide question was assigned a pre-determined domain name. A few additional domains were added to the codebook representing cross-cutting themes that had emerged during research team debriefs following the interviews. In the early stages of analysis, two coders who were part of the data collection team reviewed a subset of the interviews (*n* = 10) to assess relevancy of domains to the data, establish ease of use, and determine missing or mislabeled domains. Small revisions to the codebook were made after the initial review. The coders also reviewed one another’s subset of transcripts to establish consistency in coding. Once consistency was established, the remainder of the transcripts were allocated evenly between the two coders. Coding was done using ATLAS.ti version 8.4.4. At the conclusion of coding, the two reviewers discussed all the interviews to interpret themes and draw conclusions.

## Results

Table 1 shows the characteristics of the 22 participating practices. The majority of practices were small with a median of 3.5 full-time equivalent providers and 3.0 primary care providers. There was a mix of practice ownership between federally qualified health centers/look-alikes, clinician-owned, and hospital/health system owned (45.5, 36.4, and 18.2%, respectively). A little less than 1/3 reported being part of an accountable care organization, while a little more than half (54.6%) had achieved NCQA Patient Centered Medical Home recognition. Practices were located in 12 states, and the majority (86.4%) were in metropolitan areas. Across the practices the median percent of non-white patients was 25.9%, the median percent of Medicaid patients was 30.0%, and the median percent of Medicare patients was 25.0%.

**Table 1:** Practice Characteristics (*n* = 22)

	Median (IQR) or Percent
Number of full-time equivalent providers	3.5 (2.6)
Number of full-time equivalent primary care providers	3.0 (2.2)
Number of full-time equivalent staff	12.0 (15.8)
Practice ownership	
Federally Qualified Health Center or look-alike	45.5%
Clinician-owned	36.4%
Hospital/Health system-owned	18.2%
Part of Accountable Care Organization	31.8%
Patient Centered Medical Home recognition	54.6%
Geographic location	
Arizona	4.5%
Florida	4.5%
Georgia	4.5%
Massachusetts	4.5%
Minnesota	4.5%
Mississippi	4.5%
New York	22.7%
Ohio	13.6%
Oregon	9.1%
South Carolina	4.5%
Texas	13.6%
Washington	9.1%
Rural-urban designation	
Metropolitan area	86.4%
Small town	9.1%
Rural area	4.6%
% non-white patients	25.9 (52.5)
% Medicaid payer	30.0 (48.8)
% Medicare payer	25.0 (28.8)

We organized the results around two timeframes, early pandemic and post-pandemic. Within the early pandemic timeframe, four themes in care delivery were identified in the analysis: (1) telehealth; (2) chronic disease management; (3) screening and preventive care; and (4) access to care and equity. We further report on the future potential of practices’ service delivery adaptations after COVID-19 as described by the practices in our study. A detailed listing of themes and representative quotes is provided in Table 2.

**Table 2:** Major themes and representative quotes from high-performing practices

Theme	Representative Quotes
Telehealth	For people that are working, now they don’t have to take off all this time. They’d need 30 min to drive to the appointment. Then they’d have to wait there for 15 min. Then, they’d have their 15-min appointment and a 15-min check-out. Then, they would have to drive back. They’d miss a whole half-day of work. Now, they can just keep working, and we can call them at different times, and they can just take a quick break to get their med checks and stuff that my staff does ahead of time. When they call into me for an appointment, they are just taking a break for a few minutes, we go through what we need to do, they hang up, and they’re back to work. So, I think it’s just easy for those factors. It’s technology that we’ve had for years. We should have been utilizing it. It’s efficiency. It’s quality of care from the patient standpoint, I think, or at least satisfaction from their standpoint. (Physician)
Chronic disease management	Our RN [registered nurse] care coordinators have been running patient lists. We’re continuing to follow diabetes. We’re continuing to follow hypertension. A lot of those things our care coordinators are routinely holding televisits with folks to do their, you know, diabetes plan, diabetes education, that type of thing. (Director of Quality)
Screening and preventive care	Yeah, so that was essentially shut down. So, as far as screening colonoscopies or mammograms, all the preventive health was down to zero; that was essentially shut down. I would say probably now in the last […] maybe two to four weeks is when we’re starting to reopen those types of pathways with our local hospital here. (Medical Director)
Access to care and equity	I think our biggest struggle with that was some of the older population that don’t, I mean, we still have patients that have flip phones. They’re like, “I can’t do that telehealth stuff. I don’t even have a smartphone.” So that was a challenge for some of those older patients. Then, trying to get a family member to help them or something. But then, nobody wanted to be there; they needed to keep their distance. So, it wasn’t easy to just get a family member. If they lived with somebody or their child or somebody was taking care of them, we were able to work something out that way. But there were a lot of patients that didn’t have the capability. And, if they did, we had a struggle walking them through it because we were on the phone trying to tell them how to do it. (Office Manager)
Primary care after COVID-19	I’ve shared this term before, but I think it’s Pandora’s box. I think it got opened. The insurance companies, Medicare and Medicaid were dragging their feet on reimbursement for it [telehealth visits], and now that that box has been opened, I think that patients are certainly going to create a fuss when they’re told they have to go back [to in-person visits]. Because many of these visits, especially in chronic care management, don’t require a physical exam, or require minimal physical exam. Most of it’s really with history and monitoring, and those kinds of things very easily can be handled remotely, with the exception of things like the vital signs and weight. But if you can come up with a solution for that, yeah, I think that it would be very hard to go back to requiring all visits to be in-person. (Physician)

### Care delivery during the pandemic

#### Telehealth

A large majority of respondents reported that visit volume was down overall and that most visits were being conducted via telehealth, representing a dramatic shift from “business as usual.” Most respondents cited common benefits to telehealth that can be broadly categorized as expanded access to care. In particular, respondents noted that telehealth visits reduced barriers to care involving transportation, travel time, work conflicts, and childcare, which resulted in much lower no-show rates for virtual appointments relative to in-person appointments. In general, interviewees felt that patients appreciated the ease of access characteristic of telehealth visits and cited this as a main motivation to maintain availability of telehealth visits post-COVID-19, pending reimbursement for such services. A small number of providers cited benefits for elderly patients acknowledging that, while technology can be challenging for this subpopulation to set up initially, telehealth is the ideal visit format for this population even in the absence of a public health crisis as it reduces their exposure to common cold and seasonal flu, among other threats to their health that accompany in-person visits to the clinic. One respondent credited telehealth for bringing people back into care who had not been seen by the practice for quite some time.

#### Chronic disease management

Interviewees reported that some of their high-risk and scared patients chose to skip care, canceling their appointments. However, practices described efforts to remotely manage their patients with chronic disease, including a combination of telehealth visits, disease registries, outreach and technical assistance, and virtual education. Home-based monitoring, when available, enhanced these efforts and was said by some to be a helpful, “almost the same,” way of managing patients relative to in-person visits. The primary disadvantage noted by respondents was that not all patients have access to these monitoring devices, though one practice was able to overcome this obstacle by mailing patients blood pressure cuffs and glucometers, and another practice contracted with a company to have home International Normalized Ratio (INR) machines delivered to their patients. On the downside, practices were less able, or not able at all, to have labs taken and were not able to make necessary referrals (e.g., eye exams for diabetics). These factors led to mixed reports on whether there had been a loss in quality. Nevertheless, ethical considerations of in-person visits and labs outweighed concern about not reaching quality goals during this time. Not surprising, changes to the way care was delivered has led some practices to explore innovations to care such as drive through labs (e.g., HbA1c, INR). Near the end of our interview period, more practices were able to have patients come in for vitals and labs, with follow-up telehealth visits to review results.

#### Screening and preventive care

In the early days of COVID-19 nearly all screening and preventive services were interrupted. Respondents commented that “everything was shut down,” and that it was “just not happening.” During this time providers were less able to order routine cancer screenings and tests (e.g., mammograms, pap screens, colonoscopies, bone density tests), and most patients were not able to get routine vaccinations (e.g., pneumococcal, Shingrix). Despite reported stoppage of preventive care, several respondents reported they had discretion in determining when telehealth could fill a gap or if a patient should be seen in-person, and when necessary, patients were scheduled to come into the clinic. As in-person appointments for routine annual physical exams and access to testing became available, several respondents reported that patients were electing to hold off, preferring to wait until fall 2020 or even 2021. Although most respondents felt the months-long pause in screening and prevention would have a negligible longer-term impact, a minority expressed real concern over the consequences of delayed detection.

#### Access to care and equity

Practices were able to continue to care for their patients during the height of the COVID-19 pandemic through a combination of telehealth and limited in-person visits. However, certain groups of patients were more difficult to reach during this time. Persons with less access to the Internet, technology, or knowledge of how to navigate technology were among those that were most challenging to engage in telehealth. In particular, the older adult population was identified by several respondents as a difficult to reach population; while they were fearful of in-person visits, they often had less access to, and experience with, technology. This was particularly troubling for some respondents who felt that seniors could potentially receive the greatest benefit from telehealth visits even beyond the pandemic. Respondents reported that patients’ family members, caregivers, and in some cases practice staff helped bridge the digital divide. In addition to seniors, those with fewer resources, who are already among the most vulnerable, tended to have less access to the Internet and technology. One respondent noted that it was challenging to reach their refugee and immigrant populations and, even when reached, a lack of language concordance between the practice providers and staff and patients was a contributor to gaps in care, along with less acumen in navigating the health care system.

### Primary care after COVID-19

Respondents considered what, if any, adaptations and innovations ushered in by COVID-19 could be beneficial even post-COVID-19 and were nearly unanimous in their opinion that telehealth is here to stay saying, “Don’t think you can put the genie back in the bottle.” Several factors were identified as determinants of ongoing telehealth including reimbursement, provider acceptance, and patient demand. Already, select practices described moving towards a “Telehealth 2.0” that incorporates lessons learned during the COVID-19 pandemic. Respondents reported the need to incorporate essential staff, such as medical assistants (MAs), more in telehealth visits (e.g., using MAs as scribes during visits), a need for better workflows (e.g., developing virtual rooming processes, screening for social determinants of health), and greater use of home-based monitoring.

## Discussion

In this study we aimed to characterize the experience of primary care practices during the early months of the pandemic. Within that timeframe, we were able to report primary care provider and staff perspectives on: the nature and benefits associated with the rapid shift to telehealth; the adaptations made with respect to the care and management of patients with chronic disease as well as with routine screening and preventive care of all patients; the significance of these factors on access to care and equity; and the future potential of emerging service delivery adaptations post-pandemic among the high-performing practices in our study.

One of the primary responses to COVID-19 among our sample was a dramatic shift to telehealth visits, and this is reflective of the nationwide push towards telehealth during the COVID-19 pandemic [7–10, 18]. Although telehealth has been around for decades, it is only now that it has emerged as a critical structure because it has been a safe and timely way of providing care while maintaining social distancing efforts. Providers and staff at the high-performing practices in our study described how they managed patients remotely during COVID-19 using a combination of strategies including maintaining safe and socially distanced access through increased use of telehealth visits, providing remote patient education, and incorporating more home-based monitoring into care. Our findings also underscore the importance of maintaining and using disease registries to identify and proactively outreach to patients, a practice which was also used by the Massachusetts General Hospital system throughout the pandemic [11]. Routine screening and testing slowed considerably, resulting in concerns about delayed detection. Patients with fewer resources, lower health literacy, and older adults were the most difficult to reach and manage during this time. These findings corroborate extant literature and shine a light on the barriers to care vulnerable populations faced during this critical time [19–22].

Consistent with one report [10], providers and staff alike reported that visit volume decreased substantially after the start of the pandemic due to practice-initiated factors, such as temporarily suspending preventive care services, as well as patient-driven factors, primarily in the form of canceled appointments for non-acute issues. This finding is also supported by a recent national study which found that overall visit volume decreased by over 20% during the initial surge of COVID-19, which was largely driven by a reduction in office-based visits [23]. In general, providers and staff in our sample liked remote care for some populations and visit types, and planned to retain it in some form beyond the pandemic. This finding adds to the growing literature on the permanency of telehealth in the delivery of routine primary care [15, 22]. Due to the timing of our interviews, sustainability plans were not yet in place; however, it is clear that practices will need support in the form of sustained payment mechanisms in order to maintain remote prevention and care capabilities without jeopardizing practice finances [24, 25].

Our findings indicate that payment reform should address not only telehealth visits, but that it should also include more robust support of home monitoring devices to make remote care accessible to all patients with chronic conditions amenable to such a care plan [13]. Emerging research on the impact of COVID-19 on primary care supports our finding that patients need enhanced support for telehealth and home-based maintenance and monitoring [26, 27], including the ability to readily exchange data between patients and providers [28]. In order to ensure equity it will be important that not only payers cover the cost of home monitoring devices but also that proactive efforts are made to reduce the digital divide among difficult-to-reach, vulnerable populations [1].

This study should be interpreted in light of its strengths and limitations. Notably, the practices that participated in these interviews were high-performing practices and may represent the best-case scenario of health care delivery during this time. These practices may have demonstrated a tremendous amount of resilience because they are high performers; however, because the COVID-19 pandemic was unprecedented, these practices may not have been better positioned to adapt than other primary care practices across the country. We also conducted our interviews during the first wave of COVID-19. As the country entered later waves of the pandemic, it is possible that practices had different things to share about the way in which they were and are able to effectively provide chronic disease management and preventive care. Our study included primary care practices dedicated to adult patient populations, and many of our findings may not be applicable to pediatric populations. Relatedly, although our study included a diverse set of practices across the country, it does not allow us to know if there are trends by region or type of practice. Despite the noted limitations, this paper is novel because it is one of only a couple qualitative papers that assessed the early pandemic response of primary care practices in great depth using a unique sample of high-performing practices from across the country.

## Conclusions

COVID-19 has ushered in a paradigm shift in the delivery of high-quality primary care. While some innovations sparked by the pandemic are sure to fade away as the threat of infection diminishes, it seems likely that some of the changes made to safely accommodate the provision of chronic and preventive care are here to stay. System-wide long-term adaptations, which will likely be driven by a more permanent shift from all in-person visits and procedures to remote care for a substantial minority of visits, have the potential to improve care delivery and better meet patient needs beyond the pandemic by incorporating population health as a focus of the health system – an effort which would require payment reform [1]. Findings from this study in combination with related emerging literature indicate that more research is needed to determine which conditions are amenable to remote care, best practices for remote care, and how to support patients’ self-care and monitoring activities at home [23]. Although an endpoint for the pandemic remains uncertain, it is undoubtable that there will continue to be new adaptations made to way in which primary care practices provide chronic disease management and preventive care. Future research should qualitatively assess primary health care in the post-pandemic period.

## Data Availability

The datasets generated and analyzed during the current study are not publicly available to maintain the privacy of participants but are available from the corresponding author upon reasonable request.

## References

[1] Bakshi S, Schiavoni KH, Carlson LC, Chang TE, Flaster AO, Forester BP (2021). The essential role of population health during and beyond COVID-19. Am J Manag Care.

[2] Kmetik KS, Skoufalos A, Nash DB (2021). Pandemic makes chronic disease prevention a priority. Popul Health Manag.

[3] Fragala MS, Kaufman HW, Meigs JB, Niles JK, McPhaul MJ (2021). Consequences of the COVID-19 pandemic: reduced hemoglobin A1c diabetes monitoring. Popul Health Manag.

[4] Song H, Bergman A, Chen AT, Ellis D, David G, Friedman AB (2021). Disruptions in preventive care: mammograms during the COVID-19 pandemic. Health Serv Res.

[5] Anderson KE, McGinty EE, Presskreischer R, Barry CL (2021). Reports of forgone medical care among US adults during the initial phase of the COVID-19 pandemic. JAMA Netw Open.

[6] Gilmer TP, O'Connor PJ, Rush WA, Crain AL, Whitebird RR, Hanson AM (2005). Predictors of health care costs in adults with diabetes. Diabetes Care.

[7] Lau J, Knudsen J, Jackson H, Wallach AB, Bouton M, Natsui S (2020). Staying connected in the COVID-19 pandemic: telehealth at the largest safety-net system in the United States. Health Aff (Millwood)..

[8] Olayiwola JN, Magana C, Harmon A, Nair S, Esposito E, Harsh C (2020). Telehealth as a bright spot of the COVID-19 pandemic: recommendations from the virtual frontlines ("Frontweb"). JMIR Public Health Surveill.

[9] Sinha S, Kern LM, Gingras LF, Reshetnyak E, Tung J, Pelzman F (2020). Implementation of video visits during COVID-19: lessons learned from a primary care practice in New York City. Front Public Health.

[10] Corlette S, Berenson R, Wengle E, Lucia K, Thomas T. Impact of the COVID-19 pandemic on primary care practices. Washington, DC: The Urban Institute; 2021.

[11] Mirsky JB, Horn DM (2020). Chronic disease management in the COVID-19 era. Am J Manag Care.

[12] Mirsky JB, Thorndike AN (2021). Virtual group visits: hope for improving chronic disease management in primary care during and after the COVID-19 pandemic. Am J Health Promot..

[13] Huff C. Preventive care still on during COVID-19: ACP Internist 2020 [Available from: https://acpinternist.org/archives/2020/11/preventive-care-still-on-during-covid-19.htm.

[14] Franzosa E, Gorbenko K, Brody AA, Leff B, Ritchie CS, Kinosian B (2021). "At home, with care": lessons from New York City home-based primary care practices managing COVID-19. J Am Geriatr Soc..

[15] Srinivasan M, Asch S, Vilendrer S, Thomas SC, Bajra R, Barman L (2020). Qualitative assessment of rapid system transformation to primary care video visits at an academic medical center. Ann Intern Med.

[16] Centers for Disease Control and Prevention (2011). Million hearts: strategies to reduce the prevalence of leading cardiovascular disease risk factors--United States, 2011. MMWR Morb Mortal Wkly Rep.

[17] Gale RC, Wu J, Erhardt T, Bounthavong M, Reardon CM, Damschroder LJ (2019). Comparison of rapid vs in-depth qualitative analytic methods from a process evaluation of academic detailing in the veterans health administration. Implement Sci.

[18] Mehrotra A, Chernew M, Linetsky D, Hatch H, Cutler D, Schneider EC. The impact of COVID-19 on outpatient visits in 2020: visits remained stable, despite a late surge in cases. New York: Commonwealth Fund; 2021.

[19] Czeisler ME, Marynak K, Clarke KEN, Salah Z, Shakya I, Thierry JM (2020). Delay or avoidance of medical care because of COVID-19-related concerns - United States, June 2020. MMWR Morb Mortal Wkly Rep..

[20] Rodriguez JA, Betancourt JR, Sequist TD, Ganguli I (2021). Differences in the use of telephone and video telemedicine visits during the COVID-19 pandemic. Am J Manag Care.

[21] Eberly LA, Kallan MJ, Julien HM, Haynes N, Khatana SAM, Nathan AS (2020). Patient characteristics associated with telemedicine access for primary and specialty ambulatory care during the COVID-19 pandemic. JAMA Netw Open.

[22] Chang JE, Lindenfeld Z, Albert SL, Massar R, Shelley D, Kwok L, et al. Telephone vs. video visits during COVID-19: safety-net provider perspectives. J Am Board Fam Med. 2021;34(6):1103–14. 10.3122/jabfm.2021.06.210186 34772766

[23] Alexander GC, Tajanlangit M, Heyward J, Mansour O, Qato DM, Stafford RS (2020). Use and content of primary care office-based vs telemedicine care visits during the COVID-19 pandemic in the US. JAMA Netw Open.

[24] Rawaf S, Allen LN, Stigler FL, Kringos D, Quezada Yamamoto H, van Weel C (2020). Lessons on the COVID-19 pandemic, for and by primary care professionals worldwide. Eur J Gen Pract.

[25] Donohue D (2020). A primary care answer to a pandemic: keeping a population of patients safe at home through chronic care management and remote patient monitoring. Am J Lifestyle Med.

[26] Razavi AC, Kelly TN, He J, Fernandez C, Whelton PK, Krousel-Wood M (2020). Cardiovascular disease prevention and implications of coronavirus disease 2019: an evolving case study in the Crescent City. J Am Heart Assoc.

[27] Gupta A, Nguyen AM, Chang JE, Lai AY, Berry C, Shelley DR. Five ways—beyond current policy—to truly integrate telehealth into primary care practices. Health Affairs Blog. 2020. Available from: https://www.healthaffairs.org/do/10.1377/hblog20200903.597561/full/. [cited 2021].

[28] Omboni S, McManus RJ, Bosworth HB, Chappell LC, Green BB, Kario K (2020). Evidence and recommendations on the use of telemedicine for the management of arterial hypertension: an international expert position paper. Hypertension..

